# The immune mediated role of extracellular HMGB1 in a heterotopic model of bladder cancer radioresistance

**DOI:** 10.1038/s41598-019-42864-w

**Published:** 2019-04-23

**Authors:** Mina Ayoub, Surashri Shinde-Jadhav, Jose Joao Mansure, Fernando Alvarez, Tanner Connell, Jan Seuntjens, Ciriaco A. Piccirillo, Wassim Kassouf

**Affiliations:** 1 Urologic Oncology Research Division, Research Institute of McGill University Health Centre, Montréal, H4A 3J1 Canada; 2 Centre of Excellence in Translational Immunology (CETI), Research Institute of McGill University Health Centre, Montréal, H4A 3J1 Canada; 3 Department of Microbiology and Immunology and Program in Infectious Diseases and Immunology in Global Health, Centre for Translational Biology, Research Institute of McGill University Health Centre, Montréal, Québec, H4A 3J1 Canada; 4 Department of Medical Physics, McGill University Health Center, Montréal, H4A 3J1 Canada

**Keywords:** Cancer microenvironment, Bladder

## Abstract

Radical cystectomy (RC) together with bilateral pelvic lymph node dissection remains the standard treatment for muscle invasive bladder cancer (MIBC). However, radiation-based treatments such as tri-modal therapy (TMT) involving maximally performed transurethral resection of bladder tumor (TURBT), radiotherapy (XRT), and a chemosensitizer represent an attractive, less invasive alternative. Nevertheless, 25–30% of MIBC patients will experience local recurrence after TMT and half will develop metastasis. Radioresistance of tumor cells could potentially be one of the causes for local recurrence post treatment. High mobility group box-1 (HMGB1) was shown to play a role in bladder cancer radioresistance through its intracellular functions in promoting DNA damage repair and autophagy. Recently, HMGB1 was found to be passively released from irradiated tumor cells. However, less is known about the involvement of extracellular HMGB1 in impairing radiation response and its exact role in modulating the tumor immune microenvironment after XRT. We identified a novel mechanism of bladder cancer radioresistance mediated by the immunological functions of HMGB1. The combination of radiation plus extracellular HMGB1 inhibition markedly improved the radiation response of tumors and resulted in marked changes in the immune landscape. Moreover, combining radiation and HMGB1 inhibition significantly impaired tumor infiltrating MDSCs and TAMs -but not Tregs- and shifted the overall tumor immune balance towards anti-tumoral response. We conclude that extracellular HMGB1 is involved in bladder cancer radioresistance through promoting pro-tumor immune mechanisms.

## Introduction

Bladder cancer ranks sixth among the most common cancers in the US and is the second most common urological cancer^1^. The 5 years mortality rate for MIBC is as high as 40% in the first five years^2^. Currently, the gold standard treatment for muscle invasive bladder cancer is RC where the bladder and adjacent structures are surgically removed and a urinary diversion is created. However, patients undergoing radical cystectomy usually experience significant morbidity related to loss of normal urinary and sexual functions after surgery which have negative impacts on their quality of life after treatment^3^.

TMT (which involves a maximally performed TURBT with XRT and a chemosensitizer) has emerged as a less invasive alternative to RC with the advantage of preserving a functional bladder^4^. However, 25–30% of patients treated with TMT will still experience local recurrence and half will develop metastases^5^. Several factors can be responsible for disease recurrence including radioresistance of tumor cells. In addition, the lack of reliable predictive markers for treatment outcomes remains problematic and prevents the selection of suitable candidates for TMT. Given these reasons, studying the mechanisms of radioresistance and the factors affecting the response of the tumor to radiation is essential in order to overcome these challenges.

Radiation therapy was shown to induce an anti-tumor immune response favorable to the establishment of anti-tumoral adaptive and innate immunity^6^. However, recent studies revealed that XRT may also enhance the infiltration of immunosuppressive cells within the tumor microenvironment and the upregulation of immune checkpoint ligands which, in turn, may result in tumor relapse^7,8^. These distinct immunological outcomes may result from early events that occur within the tumor microenvironment at the time of radiotherapy.

One of the known early signals that occur following radiotherapy is the passive release of the alarmin protein High mobility group box-1 (HMGB1) from tumor cells^9^. Local dendritic cells were shown to respond to HMGB1 by enhancing antigen processing and presentation of tumor specific antigens to T cells^10^. Concomitantly, HMGB1 promotes the proliferation, survival, and function of several pro-tumor immunosuppressive cells including Regulatory T cells (Tregs)^11,12^, Myeloid Derived Suppressor Cells (MDSCs)^13^, and Tumor Associated Macrophages (TAMs)^14^. It remains to be determined how the modulation of the tumor immune microenvironment by radiation-induced HMGB1 is ultimately dictating the response to radiation.

We have previously demonstrated that intracellular HMGB1 is implicated in bladder cancer radio-resistance through its intracellular role in DNA damage repair and enhancing autophagy^15^. However, the role of extracellular HMGB1 in bladder cancer radio-resistance has never been studied. In the current study, we hypothesized that XRT-induced HMGB1 in the microenvironment of bladder cancer dictates the nature of the immune response and the outcome of the tumor.

Our novel findings demonstrate that extracellular HMGB1 is involved in immune-mediated radio-resistance of bladder cancer. The specific inhibition of extracellular HMGB1 with glycyrrhizin (GLZ) improved radiation response in tumors by the attenuation of recruitment of MDSCs and TAMs and shifting the tumor immune microenvironment towards anti-tumoral response. Targeting HMGB1 could serve as a novel therapeutic approach to overcome radiation resistance in bladder cancer.

## Results

### Radiation induces HMGB1 expression and release in bladder cancer

Radiation is known to induce cell death and the release of damage associated molecular pattern (DAMP) proteins including HMGB1. In order to study the role of extracellular HMGB1 in bladder cancer radio-resistance, we first validated MB49 cell line expression and release of HMGB1 after radiation both *in-vitro* and *in-vivo*.

**Figure 1:**
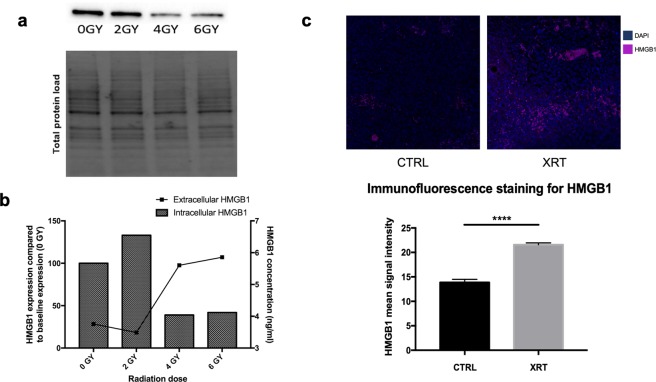
HMGB1 expression and release from the MB49 bladder cancer model *in*-*vitro* and *in-vivo*. (**a**) Western blot for intracellular fraction of HMGB1 from MB49 irradiated cells at different doses of radiation. The full gel and blot images are included in the supplementary data. (**b**) Western blot quantification bar graphs showing percentages of intracellular HMGB1 compared to non-irradiated cells; the bars represent the percentage of HMGB1 expression compared to baseline (0 GY) whereas the line is showing extracellular levels of HMGB1 in the conditioned media of cultured MB49 upon exposure to different doses of radiation. (**c**) *Ex-vivo* Immunofluorescence staining for HMGB1 showing higher levels of expression in the irradiated tumors compared to control. The bar graph is demonstrating the mean signal intensity for the staining in both groups (n = 10, single experiment, Mann-Whitney test). ****P < 0.0001.

*In-vitro*, we found that MB49 cells express HMGB1 at baseline (0 GY) as well as after radiation of the cells with increasing doses of radiation (2, 4 and 6 GY) (Fig. 1a) (Supplemantary Figure 1). Furthermore, HMGB1 was detected in the conditioned media of cultured MB49 cells and its level considerably increased with higher irradiation doses. (Fig. 1b) is summarizing the quantification of the intracellular expression and extracellular release of HMGB1 which validates our model. Moreover, these results are consistent with previous studies^16,17^ and indicates that radiation induces HMGB1 release from MB49 cells *in-vitro*.

Next, we evaluated the effects of radiation on HMGB1 expression *in-vivo*. Bladder cancer tumors developed after injection of MB49 cells into C57BL/6 mice were collected and stained for HMGB1 by immunofluorescence. We compared the expression of HMGB1 in the irradiated group vs. the control group. We found that the expression levels of HMGB1 in the irradiated tumors were significantly elevated compared to the non-irradiated tumors (P-value = 0.001) (Fig. 1b), suggesting that radiation induces the expression of HMGB1 in MB49 tumors *in-vivo*.

### HMGB1 inhibition improves radiation response of bladder cancer and hinders tumor growth post radiation *in-vivo*

We then tested the hypothesis that HMGB1 is involved in bladder cancer radio-resistance through its extracellular functions. Several compounds were shown to inhibit the release of HMGB1 or to bind directly to extracellular HMGB1 inhibiting its receptor mediated intracellular signals^18^. Specifically, Glycyrrhizin (GLZ) has the advantage of inhibiting both the extracellular release of HMGB1 as well as the interaction between HMGB1 and its cell surface receptors by specifically binding to both box domains of HMGB1^19,20^. This strategy for HMGB1 inhibition ensures near complete elimination of actively and passively released HMGB1 within the tumor microenvironment.

**Figure 2:**
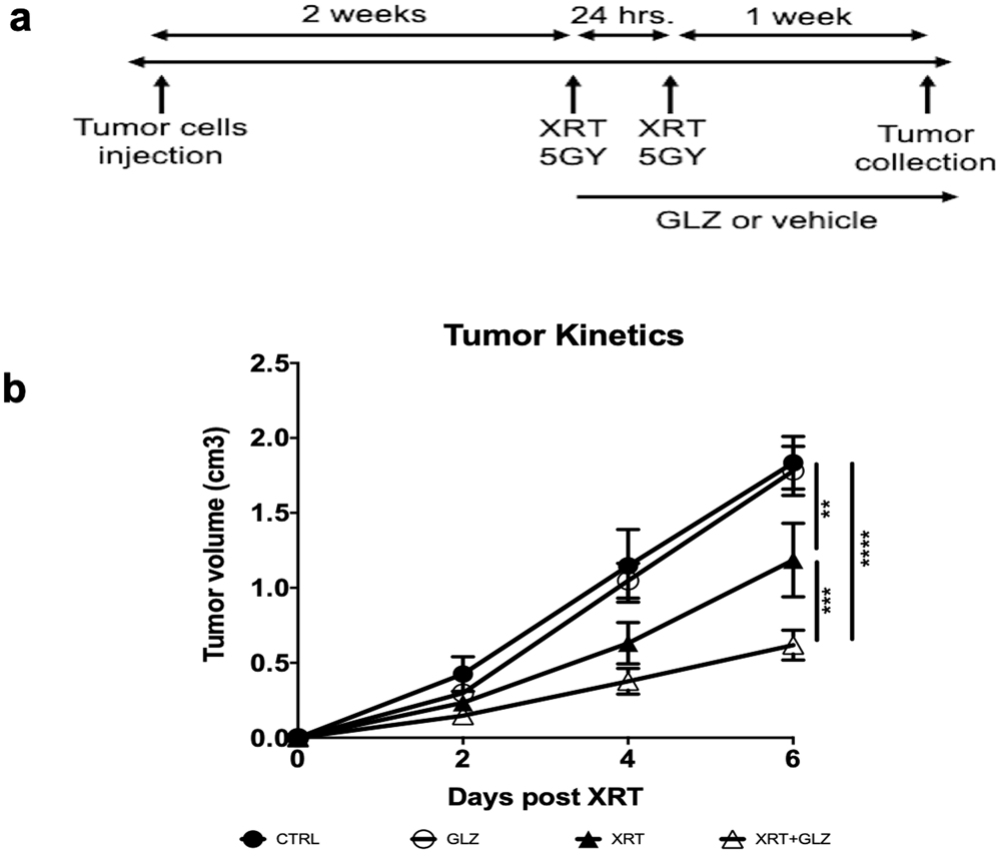
Extracellular HMGB1 inhibition results in improved radiation response of bladder cancer *in-vivo*. (**a**) *In-vivo* experiment timeline showing treatment schedule and tumor collection end points. (**b**) Tumor kinetics graph showing tumor growth rate for each of the four groups (n = 10 mice/group, the experiment was repeated 3 times). **P ≤ 0.01, ***P < 0.001, ****P < 0.0001.

MB49 cells were injected in C56BL/6 mice which were randomized into four groups: control (CTRL); glycyrrhizin alone (GLZ); radiation alone (XRT) and radiation + glycyrrhizin (XRT + GLZ). The timeline of the experiment as well as the treatment schedule are shown in (Fig. 2a). Tumor growth was monitored by serial caliper measurements and mice were sacrificed 7 days post treatment.

We noticed a similar growth pattern in the control and GLZ groups (1.83 ± 0.18 cm^3^ in CTRL and 1.78 ± 0.16 cm^3^ in GLZ). However, a significant improvement in the radiation response of the tumors was observed in the combination group compared to radiation alone (0.62 ± 0.1 cm^3^ vs. 1.19 ± 0.25 cm^3^ respectively, *P-value* = 0.001) (Fig. 2b).

These results demonstrate that extracellular HMGB1 plays a key role in bladder cancer radio-resistance and the combination of radiation and HMGB1 inhibition results in an improved response to radiotherapy.

### HMGB1 inhibition modulates bladder cancer immune landscape post-radiation

We then sought to investigate the mechanisms by which extracellular HMGB1 is mediating radioresistance of bladder cancer. Radiation is known to induce immunogenic cell death through the release of DAMP proteins including HMGB1^21,22^. Nevertheless, the release of DAMP proteins in response to radiation was shown to stimulate several immunosuppressive pathways and promote tumor immune escape mechanisms potentially contributing to the development of tumor radio-resistance^7,23^.

**Figure 3:**
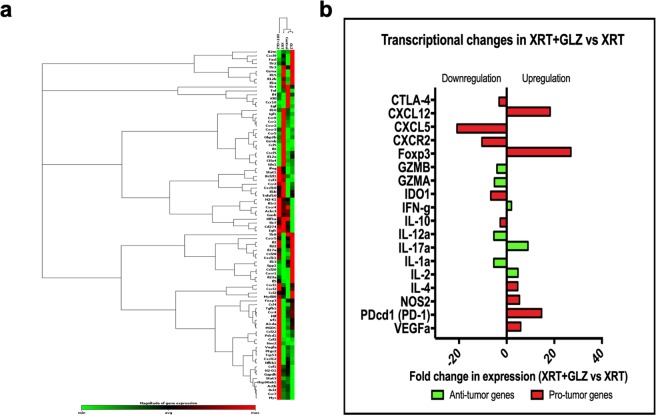
Changes in gene expression levels of certain immune related genes within the tumor immune microenvironment. (**a**) Heat map showing the differential gene expression of a set of immune related genes between groups. (**b**) Graph showing differential gene expression between XRT group and XRT + GLZ (fold change in gene expression ≥ 2 folds), red bars representing pro-tumor genes and green bars representing anti-tumor genes. Fold changes below zero indicating a downregulation of expression in the combination group compared to radiation group.

We questioned whether the release of HMGB1 in response to radiation was associated with changes in the tumor immune landscape and more importantly whether its involvement in tumor radioresistance was immune mediated. We performed a qRT-PCR array on tumor tissues collected at 1 week post radiation, evaluating a set of immune related genes in order to identify the differences in expression of these genes across our experimental groups. The set of genes included chemokines, immune-stimulatory factors, immune-suppressive factors as well as pro-inflammatory and immune inhibitory cytokines. (Fig. 3a) illustrates the complete set of genes that were studied.

Our results reveal marked differences in mRNA expression between the XRT group and XRT + GLZ group. (Fig. 3b) demonstrates genes involved in tumor immune response that showed statistical significant difference in expression between the XRT and XRT + GLZ group where statistical significance was defined as ≥2 fold change in expression between groups. Interestingly, CXCL5, a chemokine known for its role in promoting the recruitment of MDSCs and TAMs towards tumor sites^24,25^ and its receptor CXCR2 in addition to the Tregs transcription factor FOXP3 were among the highly differentially expressed between the XRT group and XRT + GLZ group (Fig. 3b).

The modulation in the tumor immune landscapes as a result of HMGB1 inhibition in the context of radiation suggested that the radiosensitization effect observed in the combination group is likely immune mediated and highlights the impaction of HMGB1 immune functions in dictating the response of the tumor to radiation therapy.

### Combining radiotherapy and HMGB1 inhibition reduces the frequency of pro-tumor MDSCs and TAMs, but not Foxp3+ Treg cells, within the tumor microenvironment

These observations lead us to investigate the effect of extracellular HMGB1 on the immune cells found in the tumor immune microenvironment. HMGB1 is known to exert cytokine-like functions on both adaptive and innate immune cells^26^. The release of HMGB1 from tumor cells in response to chemotherapy or radiation therapy was found to activate antigen presenting cells such as dendritic cells and to induce their priming of T cells involved in anti-tumor immune response^10,27^. On the other hand, tumor-derived HMGB1 has been shown to enhance the immunosuppressive capacity of Tregs^11,12^ and promote the recruitment, proliferation, and immune inhibitory functions of MDSCs^13,28,29^ and TAMs^30,31^.

**Figure 4:**
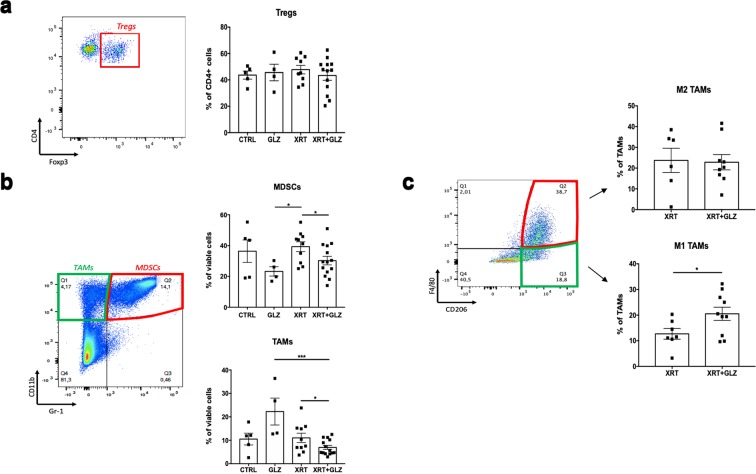
The combination of radiation and GLZ results in decreased frequency of tumor infiltrating MDSCs and TAMs at 1 week post radiation. (**a**) Gating strategy and quantification bar graphs of Tregs percentages in CD4 cells (mean ± SEM, 2 experiments, Mann–Whitney test), Tregs were identified as CD4^+^ Foxp3^+^ (**b**) Gating strategy and quantification bar graphs of MDSCs and TAMs percentages of live cells (mean ± SEM, 2 experiments, Mann–Whitney test), MDSCs were identified as CD11b^+^ Gr-1^+^, TAMs were identified as CD11b^+^ Gr-1^−^. (**c**) Gating strategy and quantification bar graphs of M1 and M2 TAMs (mean ± SEM, single experiment, Mann–Whitney test), M1 and M2 were gated from TAMs cells and identified as F4/80 + CD206- or F4/80 + CD206 + respectively. *P < 0.05, **P < 0.01, ***P < 0.001.

Tumors were collected at 1 week post radiation and then stained for flow cytometric analysis. Our results showed no significant differences in the percentage of CD4^+^ Foxp3^+^ Tregs in total CD4 cells between the XRT group and XRT + GLZ (48% ± 3.2 vs. 43% ± 3.61 respectively, P-value = 0.396) (Fig. 4a). Interestingly, the percentages of CD11b^+^ Gr-1^+^ MDSCs and CD11b^+^ Gr-1^−^ TAMs were significantly decreased in the combination group compared to radiation alone (30% ± 2.8 vs. 39% ± 3.2 respectively for MDSCs, P-value = 0.022 and 7% ± 0.9 vs. 11% ± 2 respectively for TAMs, P-value = 0.024) (Fig. 4b).

Tumor infiltrating macrophages can be further subdivided into M1 macrophages (identified as F4/80^+^ CD206^−^) which exhibit anti-tumor characteristics and M2 macrophages (identified as F4/80^+^ CD206^+^) that adopt an immunosuppressive phenotype and promote tumor progression^32^. We observed a significant increase in the frequency of anti-tumor M1 TAMs in the combination group compared to radiation alone (21% ± 2.58 vs. 13% ± 2.09, P-value = 0.04) while no significant difference was observed in the frequency of M2 TAMs (Fig. 4c).

Taken together, we demonstrate that the combination of radiation and HMGB1 inhibition results in attenuation of pro-tumor immune mechanisms within the tumor immune microenvironment by decreasing the frequency of immunosuppressive cells.

### Combining radiation with HMGB1 inhibition results in a shift in the tumor immune microenvironment towards anti-tumor immune responses

We then examined the anti-tumor immune responses developed in the tumors in order to determine how the immune balance within the tumor immune microenvironment is affected by radiation and HMGB1 inhibition.

**Figure 5:**
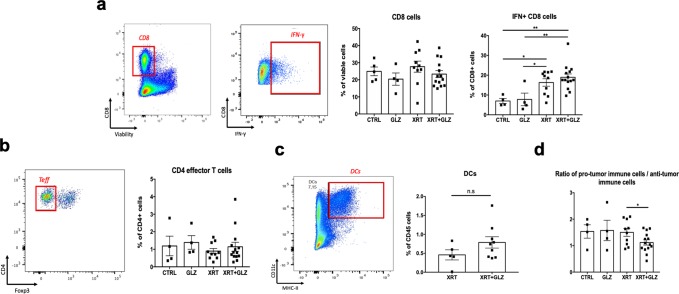
Radiation plus GLZ shift the tumor immune microenvironment towards more anti-tumor response. (**a**) Gating strategy and quantification bar graphs for the percentages of CD8 in live cells and IFN^+^ CD8^+^ cells (mean ± SEM, 2 experiments, Mann–Whitney test). (**b**) Gating strategy and quantification bar graphs for the percentages of CD4^+^ Foxp3^−^ cells effector T cells in CD4^+^ cells (mean ± SEM, 2 experiments, Mann–Whitney test). (**c**) Gating strategy and quantification bar graphs for the percentages of dendritic cells in CD45^+^ cells; (mean ± SEM, single experiment, Mann–Whitney test), dendritic cells were identified as MHCII^+^ CD11c^+^ from CD45^+^ cells. (**d**) Quantification bar graph showing the ratio of the total immunosuppressive (IS) cells (Tregs + MDSCs + TAMs) to total anti-tumor immune cells (CD4^+^ effector cells and IFN^+^ CD8^+^ cells) *P < 0.05, **P < 0.01, ***P < 0.001.

As shown in (Fig. 5a,b), no significant differences were observed in the frequencies of Foxp3^−^ CD4^+^ effector T cells and CD8^+^ cells. On the other hand, the frequency of IFN-γ^+^ CD8^+^ cells was significantly elevated in the irradiated compared to the non-irradiated groups indicating a role for radiation in enhancing anti-tumor immune responses. The frequency of tumor infiltrating dendritic cells did not change in the combination group compared to radiation alone (Fig. 5c).

We then calculated the ratio of pro-tumor immune cells (MDSCs, TAMs and Tregs) to anti-tumor immune cells (IFN-γ^+^ CD8^+^ and Foxp3^−^ CD4^+^) as an indicator for the overall immune balance within the tumor microenvironment. As expected, the combination group had a significantly lower ratio compared to the other groups (1.5 ± 0.15 vs. 1.1 ± 0.1 respectively, P-value = 0.029) suggesting a shift in the overall tumor immune balance towards an anti-tumor response (Fig. 5d).

## Discussion

TMT is a less invasive alternative to RC for the management of MIBC^4^. However, local recurrence of the disease after treatment still represents a challenge. The efficacy of TMT can be optimized by targeting tumor mediated mechanisms of radioresistance. The identification of novel pathways of resistance will allow the development of new radiosensitization therapies for bladder cancer and will improve the radiation response of the tumor.

Intracellular HMGB1 was shown by our group and others to be involved in radioresistance of several cancer types^15,16,33,34^, whereas the role of extracellular HMGB1 in radio-resistance remained unclear. In this study, we investigated the effect of extracellular HMGB1 inhibition using a known inhibitor for extracellular HMGB1, GLZ^19,20^, on the radiation response of bladder cancer tumors in a syngeneic mouse model. We observed that the combination of radiation with inhibition of the extracellular HMGB1 using GLZ resulted in a two folds reduction in tumor volume compared to radiation alone. As expected, there was no difference in tumor growth between the GLZ alone group compared with controls.

Our strategy for HMGB1 inhibition was to block the extracellular functions and the interaction of HMGB1 with its known receptors, while not interfering with its intrinsic functions within cancer cells on DNA damage repair^35^ and autophagy^36^. GLZ as an inhibitor for HMGB1 has the advantage of directly binding to both box domains of HMGB1 preventing it from interacting with its receptors^19^ and also inhibiting the phosphorylation of HMGB1 and its active secretion outside the cell^20^. We selected GLZ as it has been extensively used in the literature as an inhibitor for extracellular HMGB1^18,37^, and more importantly, has not shown any toxicity or adverse effects in clinical settings when evaluated for the treatment of liver diseases^38,39^.

In order to unravel the mechanisms behind the radiosensitization effect of HMGB1 inhibition in bladder cancer, we hypothesized that extracellular HMGB1 was mediating radioresistance through its immunological functions. As a member of the DAMP family of proteins we anticipated a role for radiation-induced HMGB1 in stimulating either pro-tumor or anti-tumor immune response within the tumor microenvironment^7,23^. Our results from the qRT-PCR array indicated major changes in the tumor immune landscape between the XRT and the XRT + GLZ group involving significant differences in expression of genes encoding for chemokines, pro-inflammatory cytokines and immunosuppressive molecules. These findings lead us to conclude that the therapeutic effects of HMGB1 inhibition are immune mediated.

Several reports have demonstrated that HMGB1 exerts many immunological functions that involve promoting the proliferation and function of MDSCs^13^, TAMs^40^ and Tregs^11^. Moreover, our qRT-PCR data showed marked change in the expression of genes encoding for CXCL5 and its receptor CXCR2 (both known for their role in the recruitment of MDSCs and TAMs^24,25^) as well the transcription factor FOXP3. Therefore, we assessed the frequency of different tumor infiltrating immune cell subsets including Tregs, MDSCs, TAMs, CD8 cells and CD4 effector T cells by flow cytometry analysis. Compared to radiation alone, GLZ supplementation during radiation therapy resulted in a significant decrease in the frequency of tumor infiltrating MDSCs and TAMs but not in Tregs. The ratio of pro-tumor immune cells to anti-tumor immune cells was significantly lower in the combination group compared to the other groups suggesting a shift in the tumor immune microenvironment towards anti-tumor immune response. These results suggested major changes in the immune profile within the tumor microenvironment mediated by the inhibition of radiation induced extracellular HMGB1.

Orthotopic bladder cancer animal models generally provide the advantage of studying tumors within their local environment. Adverse effects of XRT on bladder function and toxicity on the adjacent structures are also better studied in orthotopic models. However, the development of orthotopic models for bladder cancer usually entails the induction of mechanical, chemical or thermal injury to the urothelium in order to facilitate tumor cells attachment and implantation^41^, which creates an inflammatory reaction that might mask the changes occurring within the tumor immune microenvironment in response to XRT and in turn compromise the interpretation of the results. On the other hand, the use of heterotopic bladder cancer models to study mechanisms of radioresistance is not uncommon^15,16,42^, as these models provide an easy and reliable tool to study new mechanisms for therapy resistance as well as understanding the tumor immune microenvironment^13,43^.

In summary, in this study we identify a novel mechanism of radioresistance in bladder cancer mediated by the immunological functions of extracellular HMGB1. Therapeutic targeting of this pathway may provide a new radiosensitization approach for bladder tumors and may result in improved outcomes for patients after radiation. Further studies are needed to validate our findings in an orthotopic model and to investigate the role of biomarkers such as serum levels of HMGB1 and tumor infiltrating immune cells as predictive markers for treatment outcomes. Clinical trials using GLZ with radiation in bladder cancer patients are needed to confirm these findings.

## Conclusions

Radiation therapy induces the release of the alarmin protein HMGB1 both *in-vitro* and *in-vivo* in a bladder cancer model. Extracellular HMGB1 is involved in radioresistance of bladder cancer as indicated by the radiosensitization effect observed after the combination of radiation and GLZ. The radiosensitization effect of HMGB1 inhibition is likely immune mediated as observed by the marked changes in the tumor immune landscape and the significant decrease in the frequency of MDSCs and TAMs when combining HMGB1 inhibition and radiation.

## Materials and Methods

### Cell line and cell culture

Murine bladder cancer cell line MB49 was a gift from Dr. Peter Black (University of British Colombia). Cells were cultured in Dulbecco’s Modified Eagle’s Medium (DMEM, Wisent) supplemented with 10% fetal bovine serum (FBS, Wisent). Cells were routinely passaged when 70–80% confluent. Frequent microscopic monitoring for bacterial contamination and morphology was done.

### Protein extraction and western blot analysis

Adherent cells were scrapped from the plate in a sterile environment and treated with RIPA buffer. BCA protein kit was used for protein quantification and 20 ug of protein was loaded on the gel after being mixed with 5x laemmli buffer and heated for 7 mins at 96 degrees. An image was taken for the total protein load on the gel before membrane transfer. Primary antibody against HMGB1 (Abcam, cat.# ab79823) was incubated with the membrane at 4 °C overnight then washed. HRP conjugated secondary antibody was added at a concentration of 1:2000 in 5% milk in TBST for 1 hour at room temperature. Membranes were then imaged using chemidoc machine (Bio-Rad, Hercules, California). Western blot results analysis and protein quantification were done using the image lab software (Bio-rad, Hercules, California). Normalization of data was done using stain free gel approach from (Bio-rad) and is performed based on the total proteins measured directly from the membrane^44^.

### ELISA

Conditioned media from irradiated cells as well as control was collected 24 hours post radiation. Extracellular HMGB1 levels in the conditioned media was quantified using an ELISA kit purchased from IBL international (Hamburg, Germany) according to the manufacturer’s instructions.

### Syngeneic bladder cancer mouse model and radiation therapy

This study was approved by the McGill University Health Centre Research Institute Research Ethics Board. C57BL/6 mice were purchased from Charles River Laboratories, Inc., and kept at the McGill University Health Center-Research Institute animal facility. Ethical approval for our animal protocol #7585 was obtained and standards of the FACC at McGill University were followed for all *in-vivo* animal experiments. All animal experiments were performed in accordance with the relevant guidelines and regulations. Total of 5 × 10^5^ MB49 cells were injected subcutaneously into the right flanks of male mice between 6–8 weeks of age. Once tumors were palpable (approximately 2 weeks post implantation), mice were randomized into four groups: Control (CTRL); glycyrrhizin alone (GLZ); radiation alone (XRT) and radiation plus glycyrrhizin (XRT + GLZ). GLZ was purchased from Sigma-Aldrich (CAS number 53956-04-0) was dissolved in warm RPMI. *In-vivo* administration of GLZ was done in the XRT + GLZ group by intraperitoneal injection of 50 mg/Kg per mouse at 1 hour before radiation delivery then 4 hours after and then once daily until the end point. GLZ group was treated similarly to XRT + GLZ except for radiation treatment. CTRL and XRT groups were treated with the vehicle at the same schedule of GLZ injection. A fractionated total radiation dose of 10 Gy (2 × 5 Gy) was delivered using X-RAD smart irradiator machine (Precision X-Ray, Inc.). Tumors were allowed to grow to a maximum volume of 2 cm^3^ (primary endpoint) and they were regularly monitored at least 3 times/week. Tumor measurements were performed using a digital caliper and tumor volume was calculated based on the formula *V* = [(length × width^2^) × (π/6)]. Experiments were repeated at least twice and similar results were obtained.

### Immunofluorescence

Sections (5 μm of thickness) from formalin fixed paraffin embedded (FFPE) tissues were deparaffinized then rehydrated using serial dilutions of ethanol in distilled H_2_O (100%, 95% and 70%). Antigen retrieval was done by boiling slides in Tris-EDTA buffer (PH 9). Blocking was performed using goat serum for 1 hour then slides were incubated overnight with rabbit anti-mouse monoclonal primary antibodies against HMGB1 (Abcam 79823, 1:250) at 4 °C. Alexa-fluor 568 anti-rabbit secondary antibody (Thermofisher, A11036) was added at a concentration of 1:200 for 1 hour at room temperature. Slides were then incubated with DAPI (Thermofisher, P10144) for 5 mins and mounted with gold-antifade then visualized under the confocal microscope. Zeiss software was used to evaluate the intensities of fluorescence between images. Images from at least 4 field views at 20x were taken for each slide in order to compare fluorescence intensities between groups.

### Tumor dissociation

Mice were sacrificed at 1 week post treatment and tumors were collected into tubes filled with RPMI medium and then chopped into small pieces. Tumor Dissociation Kit (Miltenyi Biotec Inc., 130-096-730) was used for tumor digestion as indicated in the protocol and tumors were dissociated using the GentleMACS Dissociator (Miltenyi Biotec Inc.). Single cell suspensions obtained from tumors were strained against 70 μm cell strainers and then treated with ACK lysis buffer (Thermofisher, A1049201) in order to eliminate red blood cells and cells were counted using an automated counting machine.

### Multi-parametric flow cytometry and cell analysis

In order to obtain optimal results, a minimum of 40 million tumor cells were blocked with anti-CD16/32 antibody then magnetically labeled using CD4 T cell isolation kit (Miltenyi Biotec Inc., 130-104-454). A minimum of 5 million CD4 positive cells were stained with CD4-APC (eBioscience, 17-0041-81) and then fixed and permeabilized using the Foxp3 staining kit (Thermofisher, 00-5523-00) and stained intracellularly with Foxp3-FITC (eBioscience, 11-5773-80). CD4 negative cells were stimulated using PMA-Ionomycin and Golgi stop then stained for surface markers CD8-BV650 (BD bioscience, 563822), Gr-1-APC-Cy7 (BD bioscience, 557661), CD11b-APC (Biolegend, 101211), CD206-BV421 (Biolegend, 141717), F4/80-BUV395 (BD bioscience, 565614) and fixed then permeabilized using the Foxp3 staining kit and stained intracellularly for IFN-γ-PE.

### RNA extraction and RT-PCR array

For RNA extraction, 25 mg tumor tissues were kept in RNA-later solution (Thermofisher scientific, AM7030) overnight at −20 degrees then RNA was extracted from these tissues using miRNeasy Mini Kit (Qiagen, 217004). After the elimination of genomic DNA from the samples, 500 ng/ul of RNA was used to construct the cDNA using RT^2^ First Strand Kit (Qiagen, 330401). PCR array 96 well plates were purchased from Qiagen (RT² Profiler™ PCR Array Mouse Cancer Inflammation & Immunity Crosstalk, PAMM-181Z). Analysis and normalization were done using software analysis provided by Qiagen. Statistical significance was set to a threshold of more than 2 folds change in expression between groups.

### Statistical analysis

The statistical analysis was done using two tailed T test (Mann-Whitney test). Graph pad prism software was used to create the graphs and calculate P-values. A P-value of <0.05 was considered statistically significant.

## Supplementary information

Supplementary Figure 1

## Data Availability

The data supporting the work described in this manuscript is available upon reasonable request.

## References

[1] DeGeorge KC, Holt HR, Hodges SC (2017). Bladder Cancer: Diagnosis and Treatment. Am Fam Physician.

[2] Ries Lag, Y. J., Keel, G. E., Eisner, M. P., Lin, Y. D. & Horner, M.-J. SEER Survival Monograph: Cancer Survival Among Adults: U.S. SEER Program, 1988–2001, Patient and Tumor Characteristics. *National Cancer Institute* (2007).

[3] Shabsigh A (2009). Defining Early Morbidity of Radical Cystectomy for Patients with Bladder Cancer Using a Standardized Reporting Methodology. European Urology.

[4] Giacalone NJ (2017). Long-term Outcomes After Bladder-preserving Tri-modality Therapy for Patients with Muscle-invasive Bladder Cancer: An Updated Analysis of the Massachusetts General Hospital Experience. European Urology.

[5] James ND (2012). Radiotherapy with or without Chemotherapy in Muscle-Invasive Bladder Cancer. New England Journal of Medicine.

[6] Jiang W, Chan CK, Weissman IL, Kim BYS, Hahn SM (2016). Immune Priming of the Tumor Microenvironment by Radiation. Trends in Cancer.

[7] Barker HE, Paget JTE, Khan AA, Harrington KJ (2015). The Tumour Microenvironment after Radiotherapy: Mechanisms of Resistance and Recurrence. Nature reviews. Cancer.

[8] Wu C-T, Chen W-C, Chang Y-H, Lin W-Y, Chen M-F (2016). The role of PD-L1 in the radiation response and clinical outcome for bladder cancer. Scientific Reports.

[9] Wang L (2016). Ionizing Radiation Induces HMGB1 Cytoplasmic Translocation and Extracellular Release. Guo ji fang she yi xue he yi xue za zhi = International journal of radiation medicine and nuclear medicine.

[10] Apetoh, L. *et al*. The interaction between HMGB1 and TLR4 dictates the outcome of anticancer chemotherapy and radiotherapy. *Immunological Reviews*. 47–59 (2007). 10.1111/j.1600-065X.2007.00573.x 17979839

[11] Wild CA (2012). HMGB1 conveys immunosuppressive characteristics on regulatory and conventional T cells. Int Immunol.

[12] Liu Z, Falo LD, You Z (2011). Knockdown of High Mobility Group Box 1 in Tumor Cells Attenuates Their Ability to Induce Regulatory T Cells and Uncovers Naturally Acquired CD8 T Cell-dependent Antitumor Immunity. Journal of immunology (Baltimore, Md.: 1950).

[13] Li J (2017). HMGB1 promotes myeloid-derived suppressor cells and renal cell carcinoma immune escape. Oncotarget.

[14] Rojas A (2016). HMGB1 enhances the protumoral activities of M2 macrophages by a RAGE-dependent mechanism. Tumour Biol.

[15] Shrivastava S (2016). The Role of HMGB1 in Radioresistance of Bladder Cancer. Mol Cancer Ther.

[16] Jiang H, Hu X, Zhang H, Li W (2017). Down-regulation of LncRNA TUG1 enhances radiosensitivity in bladder cancer via suppressing HMGB1 expression. Radiation Oncology.

[17] Pasi F, Paolini AA-O, Nano R, Di Liberto R, Capelli E (2014). Effects of single or combined treatments with radiation and chemotherapy on survival and danger signals expression in glioblastoma cell lines. Biomed Res Int.

[18] Musumeci D, Roviello GN, Montesarchio D (2014). An overview on HMGB1 inhibitors as potential therapeutic agents in HMGB1-related pathologies. Pharmacology & Therapeutics.

[19] Mollica L (2007). Glycyrrhizin Binds to High-Mobility Group Box 1 Protein and Inhibits Its Cytokine Activities. Chemistry & Biology.

[20] He S-J (2017). The dual role and therapeutic potential of high-mobility group box 1 in cancer. Oncotarget.

[21] Golden EB, Apetoh L (2015). Radiotherapy and Immunogenic Cell Death. Seminars in Radiation Oncology.

[22] Galluzzi L, Kepp O, Kroemer G (2013). Immunogenic cell death in radiation therapy. Oncoimmunology.

[23] Hernandez C, Huebener P, Schwabe RF (2016). Damage-associated molecular patterns in cancer: A double-edged sword. Oncogene.

[24] Najjar YG (2017). Myeloid derived suppressor cell subset accumulation in renal cell carcinoma parenchyma is associated with intratumoral expression of IL-1β, IL-8, CXCL5 and Mip-1α. Clinical cancer research: an official journal of the American Association for Cancer Research.

[25] Wang G (2016). Targeting YAP-dependent MDSC infiltration impairs tumor progression. Cancer discovery.

[26] Li G, Liang X, Lotze MT (2013). HMGB1: The Central Cytokine for All Lymphoid. Cells. Frontiers in Immunology.

[27] Apetoh L (2007). Toll-like receptor 4-dependent contribution of the immune system to anticancer chemotherapy and radiotherapy. Nat Med.

[28] Li W (2013). HMGB1 recruits myeloid derived suppressor cells to promote peritoneal dissemination of colon cancer after resection. Biochemical and Biophysical Research Communications.

[29] Parker K (2014). HMGB1 enhances immune suppression by facilitating the differentiation and suppressive activity of myeloid-derived suppressor cells. Cancer research.

[30] Zhang Q-B (2016). High-mobility group protein box1 expression correlates with peritumoral macrophage infiltration and unfavorable prognosis in patients with hepatocellular carcinoma and cirrhosis. BMC Cancer.

[31] Zhang W, Tian J, Fau - Hao Q, Hao Q (2014). HMGB1 combining with tumor-associated macrophages enhanced lymphangiogenesis in human epithelial ovarian cancer. Tumour Biol.

[32] Yang L, Zhang Y (2017). Tumor-associated macrophages: from basic research to clinical application. Journal of Hematology & Oncology.

[33] Luo J, Chen J, He L (2015). mir-129-5p Attenuates Irradiation-Induced Autophagy and Decreases Radioresistance of Breast Cancer Cells by Targeting HMGB1. Medical Science Monitor: International Medical Journal of Experimental and Clinical Research.

[34] Zhao Y (2018). Wnt signaling induces radioresistance through upregulating HMGB1 in esophageal squamous cell carcinoma. Cell Death & Disease.

[35] Yuan F, Gu L, Guo S, Wang C, Li G-M (2004). Evidence for Involvement of HMGB1 Protein in Human DNA Mismatch Repair. Journal of Biological Chemistry.

[36] Tang D (2010). Endogenous HMGB1 regulates autophagy. The Journal of Cell Biology.

[37] Smolarczyk R (2012). The role of Glycyrrhizin, an inhibitor of HMGB1 protein, in anticancer therapy. Arch Immunol Ther Exp (Warsz).

[38] Li J-Y, Cao H-Y, Liu P, Cheng G-H, Sun M-Y (2014). Glycyrrhizic Acid in the Treatment of Liver Diseases: Literature Review. BioMed Research International.

[39] Hung, C.-H. *et al*. A Randomized Controlled Trial of Glycyrrhizin Plus Tenofovir vs. Tenofovir in Chronic Hepatitis B with Severe Acute Exacerbation. *Clinical And Translational**Gastroenterology* **8**, e104, 10.1038/ctg.2017.29 (2017). 10.1038/ctg.2017.29 PMC5518952 28662023

[40] Huber, R. *et al*. Tumour hypoxia promotes melanoma growth and metastasis via High Mobility Group Box-1 and M2-like macrophages. *Scientific Reports* **6**, 29914, 10.1038/srep29914 (2016). 10.1038/srep29914 PMC4947927 27426915

[41] Chan E (2009). Mouse orthotopic models for bladder cancer research. BJU Int.

[42] Wang F (2018). Chloroquine Enhances the Radiosensitivity of Bladder Cancer Cells by Inhibiting Autophagy and Activating Apoptosis. Cellular Physiology and Biochemistry.

[43] Muroyama Y (2017). Stereotactic Radiotherapy Increases Functionally Suppressive Regulatory T Cells in the Tumor Microenvironment. Cancer immunology research.

[44] Gürtler A (2013). Stain-Free technology as a normalization tool in Western blot analysis. Analytical Biochemistry.

