# Supplementary material for: The immune mediated role of extracellular HMGB1 in a heterotopic model of bladder cancer radioresistance
Source: Sci Rep. 2019 Apr 23;9:6348. doi: 10.1038/s41598-019-42864-w (PMC6478679; doi:10.1038/s41598-019-42864-w)
Supplement: Supplementary file 1 — Supplementary Figure 1 [file 41598_2019_42864_MOESM1_ESM.docx]

**Supplementary material**

**The immune mediated role of extracellular HMGB1 in a heterotopic model of bladder cancer radioresistance**

Mina Ayoub^1^, Surashri Shinde-Jadhav^1^, Jose Joao Mansure^1^, Fernando Alvarez^2,3^, Tanner Connell^4^, Jan Seuntjens^4^, Ciriaco A. Piccirillo^2,3^, Wassim Kassouf ^1*^

^1^ Urologic Oncology Research Division, Research Institute of McGill University Health Centre, Montréal, H4A 3J1, Canada.

^2^ Centre of Excellence in Translational Immunology (CETI), Research Institute of McGill University Health Centre, Montréal, H4A 3J1, Canada.

^3^ Department of Microbiology and Immunology, Montréal, H4A 3J1, Canada.

^4^ Department of Medical Physics, McGill University Health Center, Montréal, H4A 3J1, Canada.

^*^ Corresponding Author. [wassim.kassouf@muhc.mcgill.ca](mailto:wassim.kassouf@muhc.mcgill.ca)

**(figure S1)** Full gel and blot images showing HMGB1 expression at different doses of radiation

**a**

**
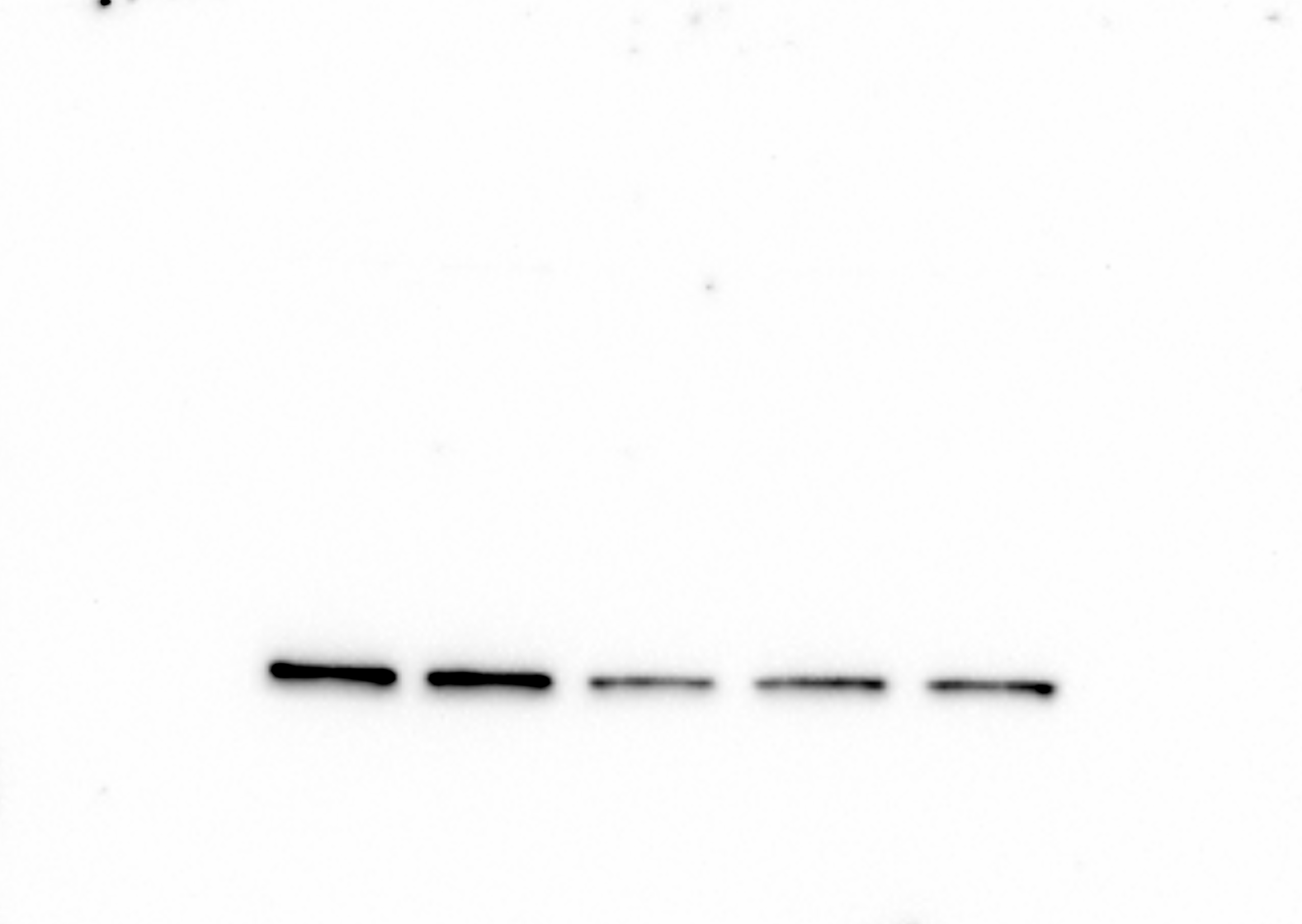

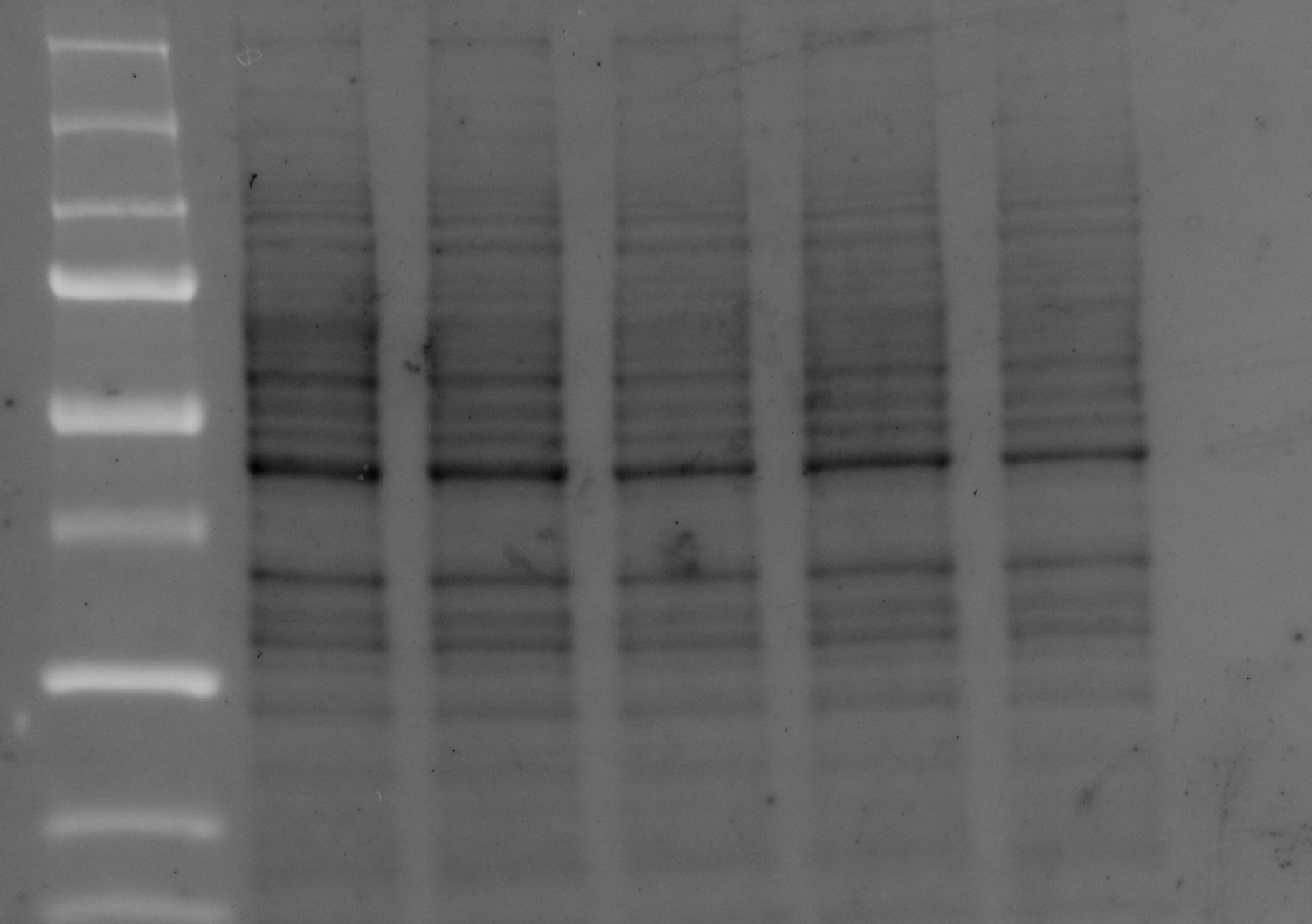
**

0GY 2GY 4GY 6GY 8GY

0GY 2GY 4GY 6GY 8GY

**b**
